# Effect of FHA and Prn on *Bordetella pertussis* colonization of mice is dependent on vaccine type and anatomical site

**DOI:** 10.1371/journal.pone.0237394

**Published:** 2020-08-21

**Authors:** Anne Zeddeman, Evi van Schuppen, Kristianne E. Kok, Marjolein van Gent, Kees J. Heuvelman, Marieke J. Bart, Han G. J. van der Heide, Joshua Gillard, Elles Simonetti, Marc J. Eleveld, Fred J. H. van Opzeeland, Saskia van Selm, Ronald de Groot, Marien I. de Jonge, Frits R. Mooi, Dimitri A. Diavatopoulos

**Affiliations:** 1 Section Pediatric Infectious Diseases, Laboratory of Medical Immunology, Radboud Institute for Molecular Life Sciences, Radboudumc, Nijmegen, The Netherlands; 2 Radboud Center for Infectious Diseases, Radboudumc, Nijmegen, The Netherlands; 3 Centre for Infectious Diseases Research, Diagnostics and Screening (IDS), National Institute of Public Health and the Environment (RIVM), Bilthoven, The Netherlands; 4 School of Biotechnology and Biomolecular Sciences, University of New South Wales Sydney, Sydney, Australia; Universidad Nacional de la Plata, ARGENTINA

## Abstract

*Bordetella pertussis* vaccine escape mutants that lack expression of the pertussis antigen pertactin (Prn) have emerged in vaccinated populations in the last 10–20 years. Additionally, clinical isolates lacking another acellular pertussis (aP) vaccine component, filamentous hemagglutinin (FHA), have been found sporadically. Here, we show that both whole-cell pertussis (wP) and aP vaccines induced protection in the lungs of mice, but that the wP vaccine was more effective in nasal clearance. Importantly, bacterial populations isolated from the lungs shifted to an FHA-negative phenotype due to frameshift mutations in the *fhaB* gene. Loss of FHA expression was strongly selected for in Prn-deficient strains in the lungs following aP but not wP vaccination. The combined loss of Prn and FHA led to complete abrogation of bacterial surface binding by aP-induced serum antibodies. This study demonstrates vaccine- and anatomical site-dependent adaptation of *B*. *pertussis* and has major implications for the design of improved pertussis vaccines.

## Introduction

The first vaccines against pertussis–a highly contagious respiratory disease primarily caused by *Bordetella pertussis—*were comprised of killed whole bacteria and introduced in the 1940s-1960s [1]. Widespread implementation of diphtheria toxoid, tetanus toxoid and whole-cell pertussis (DTwP) combination vaccines significantly reduced pertussis morbidity and mortality [2]. However, DTwP vaccines also had significant side effects and showed variable vaccine effectiveness, ranging from excellent to almost completely ineffective [3, 4]. These factors led to decreased vaccine acceptance and even temporary cessation of pertussis vaccination in some countries [5]. Consequently, less reactogenic acellular pertussis combination vaccines were developed (DTaP), comprised of 1–5 purified pertussis antigens (DTaP), i.e. pertussis toxin (PT), filamentous hemagglutinin (FHA), pertactin (Prn) and fimbriae (Fim2 and Fim3). Most high-income countries switched to DTaP vaccines from the 1990s onwards, whereas the majority of low- and middle-income countries still use DTwP vaccines [6].

The recent emergence of strains that do not express Prn (Prn^-^ strains) [7–12] has raised concern. Selective pressure by DTaP vaccines may have driven the expansion of Prn^-^ strains, which reach prevalences of 78–85% in some regions [13–15]. Although Prn^-^ strains were not attenuated compared to Prn^+^ strains in naïve mice, Prn^-^ strains did have a selective advantage compared to Prn^+^ strains in aP-vaccinated mice [16]. Interestingly, in a mixed infection model, Prn^-^ strains outcompeted Prn^+^ strains in aP-vaccinated mice, which was completely reversed in naïve mice [17]. In this study, we investigated immune selective pressure induced by DTaP and DTwP vaccines on recent Prn^+^ and Prn^-^ clinical isolates in mice.

## Material and methods

### Bacterial strains and growth conditions

*Bordetella pertussis* strains positive (B1865 and B1917, isolated in the Netherlands in 2000) or negative (B3621 and B3629, isolated in France in 2008 and 2009, respectively) for Prn expression were used for mouse challenge [18]. Inoculation stocks were prepared by growing *B*. *pertussis* in chemically defined THIJS medium under non-modulating conditions, as previously described [19, 20]. Bacteria were harvested at mid-low growth phase (OD620 0.5–0.6) and stored at -70°C.

### *B*. *pertussis* vaccination and infection

Animal experiments were approved by the Radboudumc Committee for Animal Ethics and conducted in accordance with the relevant Dutch legislation. Naïve mice were anesthetized and challenged intranasally with the different *B*. *pertussis* strains described above. For vaccination experiments, naïve mice were immunized twice with 3-week intervals by subcutaneous injection with DTaP2, DTaP3, or DTwP. Vaccinated mice were then challenged three weeks after the final dose as described above. Nasal and lung bacterial load were determined on day 3, 7, and 14 after challenge as described previously [21]. The area under the curve (AUC) was calculated for the bacterial load for each vaccine and challenge strain, using the trapezium method [22]. The length of the *fhaB* G-tract of the bacterial population after infection was determined using *fhaB* PCR. Prn mutations were verified by PCR before and after passage through the mouse as described previously [15]. Detailed procedures can be found in the Materials and methods section of the Supporting Information.

### *fhaB* phase variation

To screen large numbers of samples, a high throughput ligase detection reaction (LDR) was adapted to the *fhaB* G-tract [23]. LDR was performed on the *fhaB* PCR product containing the homopolymeric G-tract. For western blotting, the inoculation stocks of the four tested *B*. *pertussis* strains and three post-challenge B3629 bacterial samples were run, blotted, and incubated with polyclonal anti-FHA serum. Antibody binding to bacteria was measured by flow cytometry following incubation with pre-challenge serum from the different treatment groups. Detailed procedures can be found in the Materials and methods section of the Supporting Information.

### Statistical analyses

Statistical analyses on the fold-differences in post-challenge CFUs and *fhaB*-G_10_ percentages to the inoculum stocks were performed using unpaired, two-tailed t-tests. Statistical analyses on the fold-differences in the binding assays were performed using Mann-Whitney U test. Correlation of FHA production and *fhaB*-G_10_ percentage was tested using Pearsons R test. Statistical analyses were performed using SPSS22 software (IBM, New York, United States) and EXCEL. Graphs were made using GraphPad Prism 5.03 (GraphPad Software, La Jolla, United States).

## Results

### Efficacy of pertussis vaccination against *B*. *pertussis* infection

**Fig 1:**
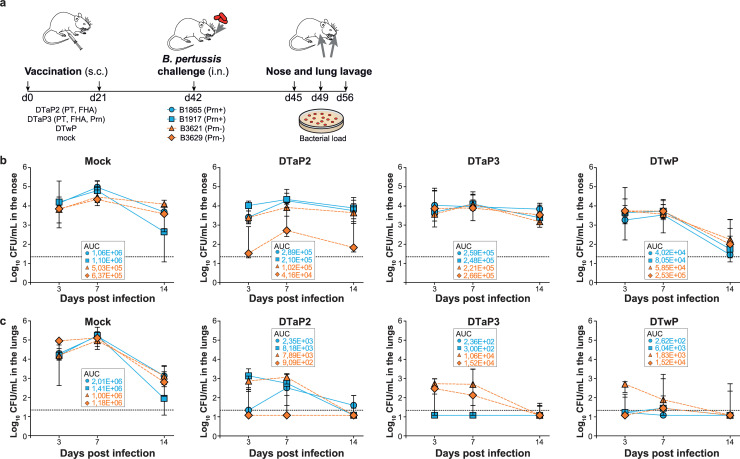
Colonization in the nose and lungs of naive and vaccinated mice. (a) Design of the study. Mice were vaccinated at day 0 (d0) and at day 21 (d21) by subcutaneous (s.c.) injection with DTaP2, DTaP3, DTwP, or mock. Three weeks after the final vaccination (d42), mice were infected intranasally (i.n.) with one of the Prn^+^ or Prn^-^ strains. Three (d45), seven (d49), and 14 (d56) days after challenge, bacterial load was determined in nose (b) and lung lavage (c). For each treatment group and challenge strain, the bacterial load over time is shown as the median log_10_ CFU ± interquartile range (n = 5–18 mice per group and time point). Dashed line indicates lower limit of detection.

To investigate vaccine efficacy against *B*. *pertussis*, groups of mice were immunized with DTaP vaccines containing either two (DTaP2; PT and FHA) or three (DTaP3; PT, FHA and Prn) pertussis components, or a DTwP vaccine containing inactivated whole *B*. *pertussis*. Unvaccinated mice were used as a control. For challenge, we performed single infections with two Prn^+^ (B1865 and B1917) and two Prn^-^ (B3621 and B3629) strains that were recently isolated from pertussis patients. All challenge strains belong to the *ptxP3* lineage, which has been highly prevalent since the 1990s in countries using DTaP vaccines [18, 24]. Apart from the Prn mutations, challenge strains were genetically nearly identical. Bacterial counts were determined in nose and lung at three, seven, and 14 days after intranasal challenge (Fig 1A). To rule out that there were major differences between bacteria recovered by lavage versus the complete bacterial pool in the nose and lungs, we compared lavage to homogenized tissue samples, showing a strong correlation between the two sampling methods (S1 Fig and Supporting Information; Material and methods for a detailed description). Bacterial loads from the challenge strains were plotted separately in Fig 1. Since the recovered bacterial load per strain was typically very low in vaccinated mice, especially in the lungs, for statistical analysis we pooled the two Prn^+^ strains, and we also pooled the two Prn^-^ stains. No statistically significant differences were observed in colonization dynamics between the Prn^+^ and the Prn^-^ pools in the nose of unvaccinated mice, suggesting that loss of Prn expression does not significantly attenuate colonization (Fig 1B). DTaP vaccination did not significantly reduce nasal colonization compared to unvaccinated mice, except on day seven when a significant reduction of Prn^-^ strains (8.5-fold, *p* = 0.04) was observed in DTaP2-vaccinated mice and a significant reduction of Prn^+^ strains (6.1-fold, *p* = 0.01) in DTaP3-vaccinated mice (Fig 1B). Minimal bacterial clearance occurred in DTaP2- and DTaP3-vaccinated mice from day seven to 14, with no significant differences in bacterial load at day 14 between vaccinated and naïve mice (Prn^+^_DTaP2: 1.4-fold, *p* = 0.6; Prn^+^_DTaP3: 1.3-fold, *p* = 0.7; Prn^-^_DTaP2: 3.7-fold, *p* = 0.4; Prn^-^_DTaP3: 5.7-fold, *p* = 0.2). Although not significant, there was a trend towards reduction in nasal colonization in DTwP-vaccinated mice on day seven (Prn^+^ strains: 23.5-fold, *p* = 0.06; Prn^-^ strains: 3.4-fold, *p* = 0.06), resulting in almost complete clearance of both Prn^+^ and Prn^-^ strains by day 14. Overall, these data suggest that none of the pertussis vaccines completely prevented infection of the upper respiratory tract, although there was a trend towards enhanced clearance in DTwP-vaccinated mice, particularly towards the later stage of infection.

Since severe pertussis is associated with progression of *B*. *pertussis* to the lungs, we determined lung bacterial load. Infection dynamics in the lungs were similar between the Prn^+^ and Prn^-^ strains in unvaccinated mice, except for day three when Prn^-^ strains reached slightly higher bacterial loads than Prn^+^ strains (2.9-fold, *p* = 0.002). This small but significant difference in mean bacterial load was mainly attributable to strain B3629 (Fig 1C). All vaccines used in this study induced significant clearance of the *B*. *pertussis* strains in the lungs at day three compared to unvaccinated controls (Fig 1C, Prn^+^_DTaP2: 22.7-fold, *p* = 0.00005; Prn^+^_DTaP3: 898.1-fold, *p*<0.00005; Prn^+^_DTwP: 559.4-fold, p<0.00005; Prn^-^_DTaP2: 115.1-fold, *p* = 0.0007; Prn^-^_DTaP3: 74.8-fold, *p* = <0.00005; Prn^-^_DTwP: 229.2-fold, *p*<0.00005), demonstrating that protection against lung infection may be achieved regardless of vaccine type and composition. Although all vaccines induced significant protection against infection in the lungs compared to naïve mice, several differences were observed between vaccines. DTaP3, which contains Prn, protected significantly better against Prn^+^ strains than DTaP2 early after challenge (39.5-fold, *p* = 0.004 at day three, Fig 1C). This was also observed for wP vaccination, which was significantly more protective against Prn^+^ strains than DTaP2 early after challenge (24.6-fold at day three, *p* = 0.04). Conversely, no significant differences were observed in vaccine efficacy against Prn^-^ strains between vaccines containing (DTwP and DTaP3) or lacking (DTaP2) Prn (Fig 1C). At day seven and 14, all vaccines significantly reduced the bacterial load of the Prn^+^ strains and Prn^-^ strains compared to unvaccinated controls (Fig 1C, Day 7: Prn^+^_DTaP2: 523.6-fold, *p* = 0.008; Prn^+^_DTaP3: 10703-fold, *p* = 0.0002; Prn^+^_DTwP: 500.9-fold, p = 0.01; Prn^-^_DTaP2: 299.1-fold, *p* = 0.007; Prn^-^_DTaP3: 84.7-fold, *p* = <0.00005; Prn^-^_DTwP: 118.9-fold, *p* = 0.001. Day 14: Prn^+^_DTaP2: 23.0-fold, *p* = 0.03; Prn^+^_DTaP3: 93.6-fold, *p* = 0.02; Prn^+^_DTwP: 150.2-fold, p = 0.02; Prn^-^_DTaP2: 163.8-fold, *p* = 0.05; Prn^-^_DTaP3: 95.3-fold, *p* = 0.01; Prn^-^_DTwP: 4.6-fold, *p* = 0.04). At day seven, DTaP3 vaccination was still more effective in clearing Prn^+^ strains compared to DTaP2 vaccination (20.4-fold, *p* = 0.0004). Of note, even though DTwP-vaccinated mice showed nearly sterilizing immunity in the lungs against all strains, a slightly better survival of one of the Prn^-^ strains (B3621) was observed early after challenge compared to Prn^+^ strains on day 3 (12.5-fold, *p* = 0.0002, Fig 1C). We also calculated the area under the curve (AUC) for each vaccine and challenge strain in the nose and lungs, which is an inverse measure of total vaccine efficacy, confirming the results described above (Fig 1B and 1C).

To observe the differences in vaccine efficacy in the lungs between Prn^+^ and Prn^-^ strains after DTaP2 and DTaP3 vaccination, a re-organized version of Fig 1B and 1C is included as a Supporting file where each panel represents the bacterial load over time per strain for the different treatment groups (S2 Fig).

### DTaP vaccination is associated with phase-variable loss of FHA expression in the lungs in Prn^-^ strains

**Fig 2:**
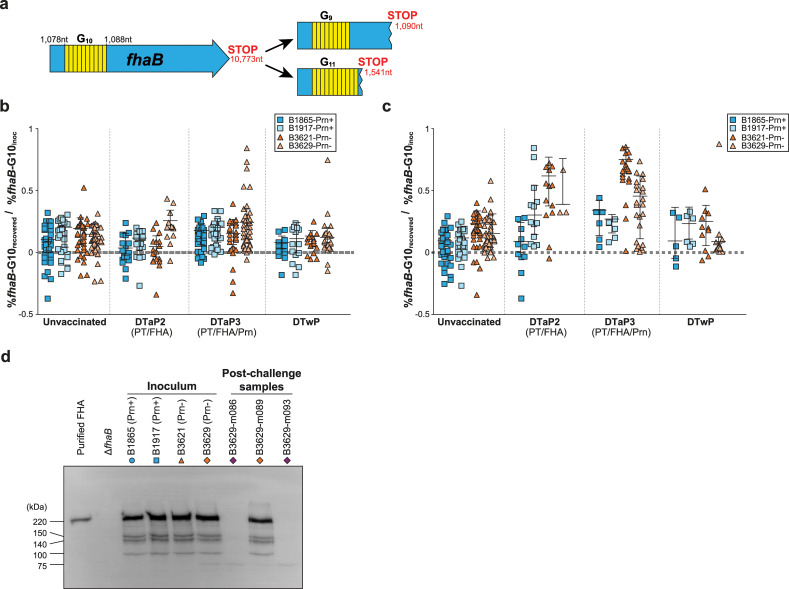
Changes in FHA expression in *B*. *pertussis* after passage through nose and lungs of naive and vaccinated mice. (a) DNA sequence of the three homopolymeric *fhaB*-G tract variants observed in *B*. *pertussis*. The wild type *fhaB* gene is 10,773 nucleotides (nt) long, with a homopolymeric tract of 10 Gs at nucleotide position 1,078–1,088. Frameshift mutations in this G-tract result in the introduction of stop codons at nucleotides 1,541 and 1,090 for *fhaB*-G_11_ and *fhaB*-G_9_, respectively. (b-c) Bacteria from the nose (b) or lungs (c) were pooled per mouse. Each symbol represents the log-transformed *fhaB*-G_10_ percentage of the recovered bacteria normalized to the *fhaB*-G_10_ percentage of its respective inoculum. Data is shown for all time points combined. Horizontal lines represent median ± interquartile range. Dashed line indicates no change in *fhaB*-G_10_ percentage of the recovered bacteria compared to its inoculum. (d) Expression of FHA in inoculum batches and in post-challenge bacterial samples was determined by western blot. Polyclonal antibodies to FHA were used; purified FHA and a *fhaB* knockout strain (Δ*fhaB*) were included as a positive and negative control, respectively. B3629-m089, B3629-m086 and B3629-m093 contain predominantly wild type, mutant, and mutant *fhaB* alleles, respectively. The position of molecular weight marker (in kDa) is shown.

Whole genome sequencing of *fhaB* in 49 clinical isolates identified phase variation in its homopolymeric G-tract. The wild type *fhaB* allele contains 10 Gs (*fhaB*-G_10_), while the two major mutant alleles contain 9 Gs (*fhaB*-G_9_) and 11 Gs (*fhaB*-G_11_) (Fig 2A). Notably, all 49 strains contained a mixture of *fhaB*-G_10_ with either *fhaB*-G_9_ or *fhaB*-G_11_. We speculate that variation in the *fhaB* G-tract would affect FHA expression. Indeed, we found a positive relationship between FHA levels and the wild type to mutant *fhaB* allele ratio in the 49 clinical strains (S3 Fig).

To assess the role of Prn and FHA in immunity against Prn^+^ and Prn^-^ strains, we studied the impact of vaccination on bacterial populations infecting the nose or lungs of mice by quantifying shifts in *fhaB* allele frequencies and bacterial loads.

For each strain we normalized the *fhaB*-G_10_ percentage of the *B*. *pertussis* population recovered from the challenged mice to that of its respective inoculum, where a value of 0 indicates a similar composition of the recovered bacteria and the inoculum. Conversely, a value higher than 0 indicates a proportional shift towards mutant *fhaB* alleles. The effect of vaccination was then assessed. Since the recovered bacterial load in the lungs was typically very low in vaccinated mice, results from the two Prn^+^ strains were pooled and results from the two Prn^-^ strains were also pooled for statistical analyses. In unvaccinated mice, no shifts in *fhaB*-G_10_ percentage were detected in the nose. A small but significant shift towards mutant *fhaB* alleles was observed in the nose of DTaP3-vaccinated mice for both Prn^+^ and Prn^-^ strains, compared to unvaccinated controls (Fig 2B, 1.6-fold, *p =* 0.046 and 1.6-fold, *p* = 0.019, respectively). This difference was mainly attributable to strain B3629, which showed a significant increase in mutant *fhaB* alleles compared to strain B3621. However, overall the *fhaB* allele composition of the recovered nasal bacterial populations was very similar to the inocula. Conversely, a strikingly different pattern emerged in the lungs. All *B*. *pertussis* populations isolated from the lungs of mice vaccinated with DTaP2 or DTaP3 showed a significant shift towards mutant *fhaB* alleles compared to unvaccinated mice (Fig 2C, Prn^+^ strains: 3.9-fold, *p =* 0.001 and 4.4-fold, *p =* 0.00005 for DTaP2 and DTaP3, respectively; Prn^-^ strains: 2.9-fold, *p =* <0.00001 and 3.1-fold, *p =* <0.00001 for DTaP2 and DTaP3, respectively). A small but statistically significant shift towards mutant *fhaB* alleles was also observed for DTwP compared to unvaccinated controls, but only for Prn^+^ strains (2.9-fold, *p* = 0.03). Intriguingly, overall increases in mutant allele percentages in the recovered bacterial populations were much more pronounced in strains lacking Prn than in Prn^+^ strains. Nonetheless, shifts towards mutant *fhaB* alleles were also observed in Prn^+^ strains in mice vaccinated with DTaP vaccines. When comparing the pooled Prn^-^ strains with the pooled Prn^+^ strains, the former had shifted significantly more towards mutant *fhaB* alleles in naïve, DTaP2-, and DTaP3-vaccinated mice (3.1-fold, *p* = 0.00001; 2.3-fold, *p* = 0.004 and 2.2-fold, *p* = 0.002, respectively), suggesting that Prn^+^ and Prn^-^ strains may differ with regards to the fitness of mutant *fhaB* alleles. Together, these results suggest that DTaP but not DTwP vaccines induce strong immune selective pressure on FHA expression in the lungs but not the nose, which is most evident in a Prn^-^ background.

To determine whether the observed shift in *fhaB* genotype correlated with FHA protein expression, western blot was performed on the recovered bacterial samples that contained predominately wild type (B3629-m089 containing 63% *fhaB*-G_10_) or mutant (B3629-m086 containing 37% *fhaB*-G_10_ and B3629-m093 containing 16% *fhaB*-G_10_) *fhaB* alleles. FHA expression in these samples was compared to the inoculum stocks. Bacterial lysates were incubated with polyclonal heat-inactivated serum from mice vaccinated with purified FHA (Fig 2D and S4 Fig). All inoculum stocks expressed FHA at the time of infection. FHA expression was detected in B3629-m089, but not in samples predominantly containing mutant *fhaB* alleles.

### Loss of recognition by DTaP-induced antibodies

**Fig 3:**
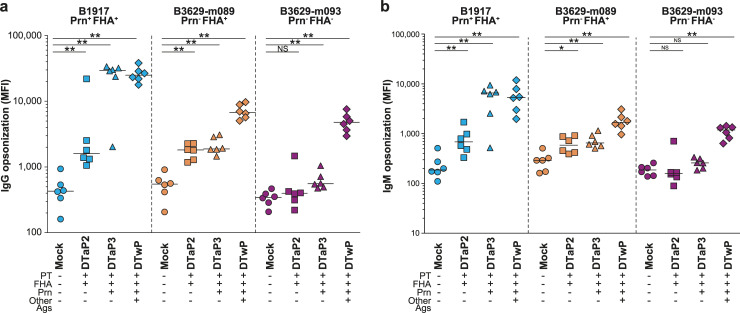
Antibody opsonization of *B*. *pertussis*. Binding of IgG (a) and IgM (b) antibodies to B1917 (Prn^+^ FHA^+^), B3629-m089 (Prn^-^ FHA^+^), and B3629-m093 (Prn^-^ FHA^-^) using 10% mouse sera from the different vaccination groups. Binding was measured using flow cytometry, MFI indicates the mean fluorescence intensity. Horizontal lines represent median. * *p*≤0.05, ** *p*≤0.01, ** *p*≤0.001.

To determine the impact of FHA and Prn non-expression on immunological recognition, antibody opsonization experiments were performed. Bacteria were opsonized with heat-inactivated post-vaccination, pre-challenge sera from vaccinated mice, after which IgG and IgM binding was determined by flow cytometry (Fig 3A and 3B). DTaP2 vaccination induced a moderate increase in IgG binding to B1917 (Prn^+^ FHA^+^) compared to unvaccinated controls (10.6-fold increase, *p* = 0.0022). Vaccination with DTaP3 and DTwP further increased IgG binding (53.1-fold, *p =* 0.0022 and 55.2-fold, *p =* 0.0022, respectively). Prn non-expression reduced binding by DTaP3-induced IgG to a level equivalent to DTaP2 (11.5-fold compared to the Prn^+^ strain, *p* = 0.0087). Prn-deficiency also affected DTwP-induced IgG opsonization levels (3.6-fold lower binding than to the Prn^+^ strain, *p* = 0.0022). The subsequent loss of FHA expression resulted in a small 1.5-fold reduction in IgG opsonization by DTwP compared to the Prn-deficient strain (*p =* 0.065). Strikingly, the combined loss of Prn and FHA expression almost completely abrogated IgG binding by either DTaP2 or DTaP3, with opsonization levels comparable to unvaccinated mice. IgM binding patterns were essentially similar to IgG (Fig 3B). These results imply a selective advantage for Prn^-^ FHA^-^ variants in vaccinated mice and possibly also humans.

## Discussion

The causes of the resurgence of pertussis have been much debated, including the relationship between pertussis vaccination and temporal changes in the *B*. *pertussis* population [25, 26]. A recent phenomenon has been the emergence and expansion of strains that have lost expression of Prn [7–9, 14, 15]. These strains have increased in frequency in countries that have implemented DTaP vaccines but not in countries using DTwP vaccines. This difference is very likely caused by the higher Prn antibody levels induced by DTaP vaccines compared to DTwP vaccines [27].Our findings demonstrate that different pertussis vaccines may exert distinct immune selective pressure on *B*. *pertussis* in the nose and lungs. We showed that *B*. *pertussis* is able to colonize the upper and lower respiratory tract. Both biotic (microbiome, receptors, immune responses) and abiotic (temperature, O_2_ pressure) factors differ at these anatomical sites. We propose that phase variation of FHA (and possibly other genes) plays a role in the adaptation of *B*. *pertussis* to the different environments it encounters in the host.

Using a mouse model, which shares many similarities to humans with regards to immunological mechanisms of *B*. *pertussis* [28], we found essentially no attenuation of Prn^-^ strains in the absence of vaccine-induced immunity, consistent with previous literature [16]. In the nose, we observed a trend towards enhanced clearance in DTwP-vaccinated mice compared to mice vaccinated with DTaP, particularly towards the late stage of infection, consistent with recent findings [29, 30].

In the lungs, all pertussis vaccines induced early clearance of *B*. *pertussis*. As expected, DTaP vaccines containing Prn were more efficacious against Prn^+^ strains than against Prn^-^ strains in the lungs early after challenge. These findings are in line with previous literature and support the hypothesis that Prn^-^ strains have emerged because they are more fit in individuals vaccinated with Prn-containing DTaP vaccines [8, 10, 15, 16, 30–32]. Indeed, pertussis patients receiving at least one pertussis vaccine dose were more likely to be infected by a Prn^-^ strain than unvaccinated patients [14]. Of note, a recent study using pertussis disease as the clinical endpoint showed that vaccine effectiveness of DTaP vaccines against Prn^-^ strains remains high [33]. An important difference with this study is that we do not have a clinical endpoint of disease due to the use of mice.

We provide evidence that FHA phase variation allows *B*. *pertussis* populations to adapt to different anatomical sites in the mouse as reflected by changes in the *fhaB* allele frequencies. Specific selection of mutant *fhaB* alleles was most prominent in the lungs of DTaP-vaccinated mice, as evident from the stronger proportional shift of Prn^-^ than Prn^+^ bacteria towards mutant *fhaB* alleles. The primary loss of Prn expression provides a selective advantage in the lungs of DTaP-vaccinated hosts, reflected in the 115-fold and 75-fold difference in bacterial load early after infection in the lungs after DTaP2 and DTaP3 vaccination, respectively. Consequently, for these strains a larger bacterial pool may be available from which bacteria bearing mutant *fhaB* alleles can be selected. FHA phase variants were only rarely detected in the nose, suggesting that FHA is essential during early colonization of the nose but not the lungs, in line with previous findings [34–37]. An alternative, not mutually exclusive explanation is that DTaP-induced immunity is simply less effective in the nose than in the lungs and therefore exerts less selective pressure in the nose. Previous human vaccination studies have also raised questions about the contribution of FHA to protection [38, 39].

Besides differences in anatomy there are also other differences in the immune system between mice and humans and consequently care should be taken to extrapolate our findings directly to humans. However, the general immunological mechanisms by which vaccines protect against pertussis have been shown to be quite similar between humans and mice [40] and studies in mice have provided important insights into immunity to *B*. *pertussis* infection. We show here that none of the vaccines completely prevent infection in the nose, but all the vaccines induced significant protection against infection in the lungs compared to naïve mice. We need further studies to confirm these findings in humans, but from our experience with the controlled human pertussis infection model [41] human volunteers who were vaccinated during infancy and who were challenged intranasally with *B*. *pertussis* can be readily colonized. At present it is unclear what the role is of FHA phase variation in humans. This study may give incentive to analyze *fhaB* phase variation in human nasopharynx and lung samples.

Using western blot, we only detected FHA expression in recovered bacterial populations that contained predominantly wild type *fhaB* alleles (B3629-m089) and not in the samples containing predominantly mutant *fhaB* alleles (B3629-m086 and B3629-m093). Although bacteria bearing mutant alleles were predicted to produce small truncated FHA molecules due to premature termination of translation, immunoblotting with anti-FHA polyclonal serum did not detect such protein fragments. Possibly, the small fragments are not produced, are degraded, are not recognized by the serum used, or the detection method is not sensitive enough to detect FHA fragments in mixed populations containing lower proportions of wild type *fhaB* alleles. It is conceivable that the FHA fragments are present, but not recognized by the antibodies raised against the native protein. Interestingly, both predicted fragments contain a two-partner secretion (TPS) domain which is essential for secretion [42]. Indeed, a 304 residue N-terminal FHA fragment, nearly identical to the 292 residue fragment predicted to be produced by *fhaB*-G_9,_ was shown to be secreted by *B*. *pertussis* [42]. Thus, it is possible that the *fhaB*-G_9_ and/or *fhaB*-G_11_ fragments are excreted and have some function. However, apart from the TPS domain, known functional domains of FHA have been located outside the predicted *fhaB*-G_9_ and/or *fhaB*-G_11_ fragments [43]. Strikingly, *B*. *pertussis* bacteria that lost expression of both Prn and FHA were not recognized anymore by DTaP-induced antibodies. With the loss of both Prn and FHA as antigenic targets for opsonization, DTaP-induced immunity against *B*. *pertussis* will therefore be mostly dependent on the recognition of PT. Although PT is predominantly a secreted protein and anti-PT antibodies do not bind to the bacterium, monocomponent pertussis vaccines based only on PT have been shown to be effective in Denmark [44]. Because Prn^-^ FHA^-^ variants were ultimately still cleared in the lungs of DTaP-vaccinated animals, the question is thus how protection is achieved. It seems likely that PT antibodies confer passive protection in mice, presumably through neutralization of the biological activity of PT [45]. In addition, cellular immunity may play a role.

Although naturally occurring FHA mutants have been described before [46] and we also identified clinical strains with mutations in the *fhaB*-G_10_ region, they have not been identified very frequently. One possible explanation for this is that the standard diagnostic sampling procedure for pertussis patients is to collect a nasopharyngeal swab. Because such samples are not obtained from the deeper airways, it is entirely possible that FHA phase variation in human pertussis patients is a frequent event that remains largely undetected.

An important question that we have not yet addressed in this study is the relation between bacterial load, disease induction and transmission. Since the Prn^-^ FHA^-^ variants were not recognized by DTaP-induced immunity in the lungs, it is likely that non-expression of both Prn and FHA as antigenic targets has a direct effect on survival of the bacteria and hence the duration, disease severity, and symptoms. The selection of FHA-negative phase variants in the lungs and reduced immune recognition of these variants in DTaP-vaccinated hosts could increase the duration of infection, the bacterial load and the severity of clinical symptoms, thereby potentially enhancing transmission of *B*. *pertussis*. Although Warfel *et al*. showed that transmission may occur even in the absence of clinical symptoms [30], it is not unlikely that infection of the lungs and the induction of coughing enhances transmission rates. This would imply that there is positive selective pressure on *B*. *pertussis* for infection of the lower respiratory tract of vaccinated individuals and the induction of clinical symptoms. Transmission is thought to occur during the catarrhal phase until up to three weeks after the start of the paroxysmal phase. Since FHA phase variation was virtually undetected in the upper respiratory tract but was commonly detected in the lower respiratory tract, it would be interesting to investigate the transmission and disease capacity of strains deficient in both Prn and FHA and the role of phase-variable antigen expression in the epidemiology of pertussis, for instance in the baboon model.

In conclusion, our study highlights the importance of studying the effects of vaccination on pathogen populations *in vivo*. Importantly, it suggests that the efficacy of DTaP vaccines against Prn^-^ strains may be further reduced due to phase variable expression of FHA. Immunity to *two* acellular pertussis vaccine components may therefore be compromised.

## Supporting information

S1 File (DOCX)

S1 Fig Correlation between bacterial populations recovered from the airways via lavage versus homogenized tissue samples from the same mouse. (a) Correlation between %*fhaB*-G_10_ of lung lavage samples and homogenized lung tissue. (b) Correlation between %*fhaB*-G_10_ of nose lavage samples and homogenized nasopharynx tissue. Dashed lines indicate 95% confidence intervals.
(EPS)

S2 Fig Colonization in the nose and lungs of naive and vaccinated mice. Mice were vaccinated at day 0 (d0) and at day 21 (d21) by subcutaneous (s.c.) injection with DTaP2, DTaP3, DTwP, or mock. Three weeks after the final vaccination (d42), mice were infected intranasally (i.n.) with one of the Prn^+^ or Prn^-^ strains. Three (d45), seven (d49), and 14 (d56) days after challenge, bacterial load was determined in nose (a) and lung lavage (b). Each panel represents the bacterial load over time per strain for the different treatment groups, shown as the median log_10_ CFU ± interquartile range (n = 5–18 mice per group and time point). Dashed line indicates lower limit of detection.
(EPS)

S3 Fig Correlation of FHA production with the percentage of *fhaB*-G_10_. The amount of FHA produced and the length of the homopolymeric G-tract was determined using 49 clinical isolates. The percentage G_10_ of each isolate was calculated by dividing the G_10_ (wild type) LDR signal by the total G_10_, G_9_, and G_11_ signal. A significant positive correlation was found (Pearson r 0.49, *p*<0.05).
(EPS)

S4 Fig Unadjusted western blot image. Original blot containing molecular weight marker and loading order of experimental samples.
(EPS)
