# Supplementary material for: Effect of FHA and Prn on Bordetella pertussis colonization of mice is dependent on vaccine type and anatomical site
Source: PLoS One. 2020 Aug 21;15(8):e0237394. doi: 10.1371/journal.pone.0237394 (PMC7446907; doi:10.1371/journal.pone.0237394)
Supplement: S1 File — (DOCX) [file pone.0237394.s001.docx]

**Supporting Information**

**Material and methods**

**Bacterial strains and growth conditions**

*Bordetella pertussis* strains positive (B1865 and B1917, isolated in the Netherlands in 2000) or negative for Prn expression (B3621 and B3629, isolated in France in 2008 and 2009, respectively) were used to infect mice [1]. All challenge strains were obtained via the Dutch National Surveillance System from patients with confirmed whooping cough. All four strains used in this study (B1865, B1917, B3621 and B3629) were isolated from patients with pertussis symptoms. The four strains belonged to the same Single Nucleotide Polymorphism Type (SNP type) as determined by SNPex [1], meaning they were closely related. Strains B3621 and B3629 had a 25 bp deletion and an IS481 insertion in *prn*, respectively. All strains carried the same alleles for FHA, the pertussis toxin A subunit, the pertussis toxin promoter, the serotype 2 fimbrial subunit and the fimbrial serotype 3 subunit (respectively, *fhaB1*, *ptxA1*, *ptxP3, fim2.1* and *fim3.2*). The two strains producing Prn harbored the *prn2* allele. Further, all four strains expressed *fim3* and not *fim2*, i.e. they were serotype 3 strains. The complete genome sequences of the four strains are available (accession numbers: B1865 NZ_CP011441, B1917 NZ_CP009751, B3621 NZ_CP011448, and B3629 NZ_CP011400). All four strains belong to the *ptxP3* lineage, which has been highly prevalent since the 1990s in countries using DTaP vaccines. Further, except for Prn, no differences were found in the DNA sequences for known virulence-associated genes. The relatedness of the four strains is also suggested by their very similar growth curves in naïve mice. Age of the patients from whom de isolates were obtained were unknown. B1865, B1917, and B3621 were obtained from unvaccinated patients and B3629 was isolated from a vaccinated patient. The challenge inocula were prepared by growing *B. pertussis* in chemically defined THIJS medium under non-modulating conditions as previously described [2, 3]. Bacteria were harvested at mid-log growth phase (OD620 0.5 - 0.6), flash frozen and stored at -70°C.

***B. pertussis* vaccination and infection**

Animal experiments were approved by the Radboudumc Committee for Animal Ethics and conducted in accordance with the relevant Dutch legislation. Upon arrival, mice were randomly distributed over individually ventilated cages. Sterile water and food were provided ad libitum. Prior to the first vaccination experiment, mice had at least one week to acclimate. The ability of strains to colonize naïve and immunized mice was investigated by infection of mice. Groups of 6 naïve, female, 6 – 8 week old, BALB/c mice (Harlan, Indianapolis, USA), were challenged intranasally with 40 µL of *B. pertussis* under isoflurane anesthetics (1.5-2.5% v/v over O_2_ after induction). Mice were infected with B1865 (9x10^4^ CFU and 6x10^6^ CFU), B1917 (5x10^7^ CFU and 7x10^6^ CFU), B3621 (8x10^4^ CFU and 8x10^6^ CFU), and B3629 (7x10^5^ CFU, 2x10^6^ CFU, and 2x10^7^ CFU). After challenge, mice were monitored daily, weighed and scored according to their condition. Terminal bleeding occurred under general anesthesia.

For vaccination experiments, groups of 6 naïve, female, 6 – 8 week old BALB/c mice (Harlan, Indianapolis, USA), were immunized twice with 3-week intervals by subcutaneous injection with 1/50^th^ human dose of Infanrix-hexa (GSK, Belgium; consisting of 25 µg PT, 25 µg FHA, and 8 µg Prn), 1/50^th^ human dose of Pentavac (Sanofi Pasteur, France; consisting of 25 µg PT and 25 µg FHA, or 1/50^th^ human dose of the Polish wP vaccine (IBSS Biomed S.A., Poland, kindly donated by Anna Lutyńska from the Polish National Institute of Public Health – National Institute of Hygiene, NIPH-NIH; containing three strains designated 606-PL, 186-Jug and 629-PL, isolated in, respectively 1977, 1965 and 1977 [4]. Vaccinated mice were challenged three weeks after the final vaccination by intranasal inoculation with *B. pertussis* in 40 µL. Mice were infected with B1865 (6x10^6^ CFU and 4x10^6^ CFU), B1917 (7x10^6^ CFU and 6x10^6^ CFU), B3621 (8x10^6^ CFU and 9x10^6^ CFU), and B3629 (2x10^6^ CFU, 2x10^7^, and 6x10^6^ CFU). Of note, we were not able to repeat infection with B3629 at the required dose, because the DTaP2 vaccine was no longer available.

The bacterial load in the nose and lungs of naïve and vaccinated mice was determined on day three, seven, and 14 after challenge as described previously [5]. Colonization was quantified by plating 10-fold serial dilutions in PBS of nose lavage (NL) and bronchoalveolar lavage (BAL) on Charcoal-horse blood plates containing cephalexin (CHB-1) (Mediaproducts, Groningen, the Netherlands). CFUs were counted after four to five days of growth at 35°C. Plates with zero colonies were considered to contain half the detection limit of 12 CFU/mL for statistical analysis. In this study, we used lavage to recover bacteria from the airways. Since bacterial populations may differ between tissue and lavage samples, we have validated the lavages compared to homogenized tissue samples, by collecting both homogenized tissue and lavage samples from a limited number of mice. This showed a very strong correlation between %G_10_ of the lung lavage compared to the homogenized lung tissue (R^2^= 0.88, S1a Fig). The correlation between %G_10_ in nose lavage and homogenized nasopharynx tissue was much less pronounced (R^2^ = 0.13, S1b Fig). There is however minimal variation between the nasopharynx samples, which could explain the lack of statistical correlation in the nose samples. Area under the curve, which is an inverse measure of vaccine potency, was calculated for the CFU curves using GraphPad Prism 5.03 (GraphPad Software, La Jolla, United States).

To determine the length of the *fhaB* G-tract of the bacteria (see below for details) after passage through the mouse, all *B. pertussis* colonies were harvested from the NPL and BAL plates in 300 µL of saline solution. For *fhaB* PCR, 50 µL of this suspension was transferred to a new tube and inactivated by heating at 95°C for 2 minutes. Glycerol was added to the remaining solution to a 15% final concentration and stored at -80°C for western blot and antibody opsonization experiments. For each of the four strains we compared the percentage of wild type *fhaB* allele *(fhaB*-G_10_) in the *B. pertussis* populations before and after infection. Since the recovered bacterial load in the lungs was typically low in vaccinated mice and the duration of infection did not have an effect on the percentage of *fhaB*-G_10_ (results not shown), the *fhaB*-G_10_ percentage data from, respectively, the two Prn^+^ and the two Prn^-^ strains were pooled for all three days. The corresponding formula is 1 – (% *fhaB-*G_10recovered_ / % *fhaB*-G_10inoculum_). A value of 0 indicates that the recovered bacterial population is identical to the inoculum (10 Gs), a value below 0 indicates that the recovered bacterial population contained more wild type *fhaB-*G_10_ than the inoculum, and a value above 0 that the recovered bacterial population contained less *fhaB-*G_10_ than the inoculum. Lastly, the mutation in Prn was verified by PCR before and after passage through the mouse as described previously [6].

**Identification of mutations in the *fhaB* homopolymeric G-tract (HGT)**

Mutations in the *fhaB* HGT were determined using Sanger sequencing. Briefly, bacteria were grown and harvested in PBS. This suspension was lysed and a PCR was performed with the supernatant. PCR primers were designed to flank a region of 700 bp containing the *fhaB* G-tract of interest. The products were sequenced with the ABI Prism 3730 DNA Analyzer (Applied Biosystems, California, USA) and analysed with CLC bio main workbench 7.5.1 software (CLCbio, Aarhus, Denmark).

**Determination of the *fhaB* G-tract length using the ligase detection reaction (LDR)**

To screen large numbers of samples, a high throughput ligase detection reaction (LDR) was adapted to the *fhaB* G-tract [7]. In the LDR, a Fluorescein amidite (FAM) labeled common primer aligns with the last 5 Gs of the homopolymeric tract and the 16 bases flanking the G-tract on the right. Subsequently, three specific primers were added, each with 4 Cs, 5 Cs or 6 Cs (to match G-tracts with respectively 9, 10 or 11 Gs) and 15 bases complementary to the sequence flanking the G-tract on the left. These specific primers have a distinct length due to a poly-A-tail. The specific primer also binds to the G-tract, and only when the common and specific primer aligns exactly, the oligo’s are joined by the ligase enzyme. This way, a fluorescent product of 49 bp (G_10_), 72 bp (G_9_), or 111 bp (G_11_) is formed that can be detected by capillary electrophoresis.

The LDR was performed on the *fhaB* PCR product containing the homopolymeric G-tract, which was generated as described in the section mouse infection experiments. The LDR mixture had a total volume of 20 µL and contained of 1 µL PCR product, 1 µL Taq DNA Ligase (New England Biolabs, Ipswich, USA), 2 µL Taq DNA ligase reaction buffer (New England Biolabs, Ipswich, USA) and 0.5 pmol of each of the common and specific oligonucleotides. The oligonucleotides were synthesized and RP-HPLC or PAGE purified by Eurogentec (Eurogentec, Maastricht, The Netherlands). The LDR mixture was heated for 1.5 minute at 94°C; followed by 30 cycles of 15 seconds at 94°C and 2 minutes at 65°C. The LDR products were diluted 40 times, and 2 µL of the dilution was mixed with 10 µL LIZ120 size standard (Applied Biosystems). The samples were run on an ABI3730xl DNA Analyzer (Applied Biosystems, California, USA) by BaseClear (BaseClear, Leiden, The Netherlands). As a positive control for fragment analysis, synthetic DNA (0.05 pmol) with 9, 10, or 11 Gs was used. The raw trace data was analysed with Genemarker 1.5.1 software (Softgenetics, Pennsylvania, USA). The percentage G_10_ was calculated by adding up the peaks of G_9_, G_10_, and G_11_ products and determining the proportion of G_10_. The peaks of products formed on the synthetic DNA were used to verify the fragment size of the *fhaB* variants.

**Protein levels of FHA**

A linear relationship between FHA production and the percentage of strains containing the *fhaB*-G_10_ allele was found using 49 strains (S2 Fig). Antigen expression was quantified indirectly using a microsphere-based Multiplex Immunoassay (Luminex, Austin, USA). FHA monoclonal antibody 29E7 was used to bind FHA and purified FHA (269 µg/ml) was used in a dilution series to determine FHA production. As control, we also determined the level of PT and Prn production by these strains. Eight of these strains were previously shown to produce low levels of FHA (<35 ng at OD600). Three other strains produced high levels of FHA (>90 ng at OD600). The remaining 38 strains were all clinical isolates received by the RIVM (National Institute for Public Health and the Environment, the Netherlands) between January and May 2014. For quantification of PT, Prn, and FHA production, bacteria were cultured for 3 days at 35°C on BG agar plates, supplemented with 15% sheep blood. Subsequently, a smear of colonies was recultured on BG agar plates for 48 hours at 37°C and harvested by suspension in 1.2 ml PBS. The OD620 of the cell suspension was used to correct for cell density differences when calculating the amount of FHA produced. The cell suspension was also used to determine the length of the G-tract in *fhaB* by PCR and LDR, as described above.

**FHA immunoblotting**

For western blotting, the inoculum stocks of the four tested *B. pertussis* strains were heat-killed for 30 minutes at 56°C. In addition, three post-challenge samples were cultured, harvested, and heat-killed. The bacterial strains were diluted in PBS to an OD_620_ of 1.25, 1x sample buffer (end concentration) was added, and heated for a minimum of 5 minutes at 95°C. Purified FHA (163.5 μg/mL) was diluted to a concentration of 1 μg/mL. Purified FHA (positive control), a *fhaB* deletion mutant (*ΔfhaB*, negative control), the inoculum strains (B1865, B1917, B3621, B3629) and the three post-infection bacterial populations (B3629-m086, B3629-m089, B3629-m093) were run on a 6% SDS-PAGE gel and blotted onto PVDF membrane. The membrane was blocked with 2% ELK (milk powder, Campina, the Netherlands) in 0.1% PBS/Tween (PBS-T), and incubated overnight at 4°C with polyclonal mouse anti-FHA (NA6677 BIG 5128 FHA Gp4; obtained from Prof. Andrew Gorringe, Public Health England, Porton Down) at 1:100 dilution. The membrane was washed three times with PBS-T, incubated with 1:5000 peroxidase-conjugated rabbit anti-mouse antibody, and developed with enhanced chemiluminescence (ECL prime kit, GE Healthcare). The membrane was exposed using the FluorChem E Digital Darkroom (Proteinsimple).

**Antibody opsonization**

Antibody deposition on B1917, B3629-m089 and B3629-m093 was measured by incubating 10^7^ CFU with 10% naïve and immunized mouse sera (heat-inactivated for 20 minutes at 56°C) in PBS + 2% BSA for 30 minutes at 37°C + 5% CO2 while shaking. Since the post-vaccination pre-challenge serum volume was too low to measure individual mouse samples, serum samples were pooled from two mice for each treatment group. Subsequently, the bacteria were washed twice with PBS to remove non-bound antibodies and incubated with 1:100 goat polyclonal α-mouse IgG-FITC (Fc-specific, Sigma) or 1:100 rat monoclonal α-mouse IgM-APC (clone II/41, BD Biosciences) in PBS + 2% BSA for 30 minutes at 4°C. The bacteria were then fixed in 2% paraformaldehyde for 20 minutes at room temperature. Surface-bound IgG and IgM was detected by flow cytometry using a FACS LSR-II (BD biosciences, SanJose, CA, USA). Data were analyzed using FlowJo Version X (FlowJo, LLC, Ashland, OR, USA).

**Statistical analyses**

Statistical analyses on the fold differences in CFUs and the *fhaB*-G_10_ percentage in *B. pertussis* to the inoculum stocks were performed using unpaired, two-tailed t-tests. Statistical analyses on the fold differences in the binding assays were performed using Mann-Whitney U test. Correlation of FHA production and percentage *fhaB*-G_10_ was tested using Pearsons R test. Statistical analyses were performed using SPSS22 software (IBM, New York, United States) and EXCEL. Graphs were made using GraphPad Prism 5.03 (GraphPad Software, La Jolla, United States).

**References**

1. Bart MJ, van der Heide HG, Zeddeman A, Heuvelman K, van Gent M, Mooi FR. Complete Genome Sequences of 11 Bordetella pertussis Strains Representing the Pandemic ptxP3 Lineage. Genome Announc. 2015;3(6).

2. Thalen M, van den IJ, Jiskoot W, Zomer B, Roholl P, de Gooijer C, et al. Rational medium design for Bordetella pertussis: basic metabolism. J Biotechnol. 1999;75(2-3):147-59.

3. de Gouw D, Hermans PW, Bootsma HJ, Zomer A, Heuvelman K, Diavatopoulos DA, et al. Differentially expressed genes in Bordetella pertussis strains belonging to a lineage which recently spread globally. PLoS One. 2014;9(1):e84523.

4. Gzyl A, Augustynowicz E, Gniadek G, Rabczenko D, Dulny G, Slusarczyk J. Sequence variation in pertussis S1 subunit toxin and pertussis genes in Bordetella pertussis strains used for the whole-cell pertussis vaccine produced in Poland since 1960: efficiency of the DTwP vaccine-induced immunity against currently circulating B. pertussis isolates. Vaccine. 2004;22(17-18):2122-8.

5. Cron LE, Stol K, Burghout P, van Selm S, Simonetti ER, Bootsma HJ, et al. Two DHH subfamily 1 proteins contribute to pneumococcal virulence and confer protection against pneumococcal disease. Infect Immun. 2011;79(9):3697-710.

6. Zeddeman A, van Gent M, Heuvelman CJ, van der Heide HG, Bart MJ, Advani A, et al. Investigations into the emergence of pertactin-deficient Bordetella pertussis isolates in six European countries, 1996 to 2012. Euro Surveill. 2014;19(33).

7. Gogol EB, Cummings CA, Burns RC, Relman DA. Phase variation and microevolution at homopolymeric tracts in Bordetella pertussis. BMC Genomics. 2007;8:122.
